# Pore-Scale Insights into the Contribution of Clays during Low-Salinity Waterflooding in Sandstones

**DOI:** 10.1021/acs.energyfuels.4c06254

**Published:** 2025-09-05

**Authors:** Edward Andrews, Ann Muggeridge, Alistair Jones, Samuel Krevor

**Affiliations:** Department of Earth Science and Engineering, 4615 Imperial College, London SW7 2AZ, U.K.

## Abstract

The presence of clay minerals is generally accepted to be a necessary condition for low-salinity flooding to improve oil recovery in sandstones. However, there have been no in situ pore-scale observations that low-salinity brine releases more oil from pores with higher proportions of clay minerals present. In fact, there is a growing body of evidence that significant oil release can occur from quartz and feldspar mineral surfaces during low-salinity flooding. In this work, we use X-ray Micro-CT imaging technology to image unsteady-state tertiary low-salinity waterflooding experiments in Berea, Bunter, and Castlegate sandstone samples. We make the first pore-scale in situ observations of oil release from clay-rich and clay-poor pores during low-salinity flooding in oil-wetting sandstone samples. We find that clay minerals are initially significantly more oil-wetting than other mineral groups in all samples. In the Bunter and Berea samples, we observe a significant wettability alteration during low-salinity flooding for clay minerals and nonclay minerals; however, no significant wettability alteration is observed in either mineral group in the Castlegate sample. In all samples, clay-poor pores contribute significantly more to total oil production during low-salinity waterflooding than clay-rich pores. Clay-rich pores are found to account for only 15, 5, and 3% of total additional recovery in the Berea, Bunter, and Castlegate samples, respectively. We see no evidence that clays dominate the response to low-salinity flooding in any of the samples. This work adds to the growing body of evidence that questions the presence of clays as a necessary condition for a favorable response to low-salinity flooding in sandstones.

## Graphical abstract

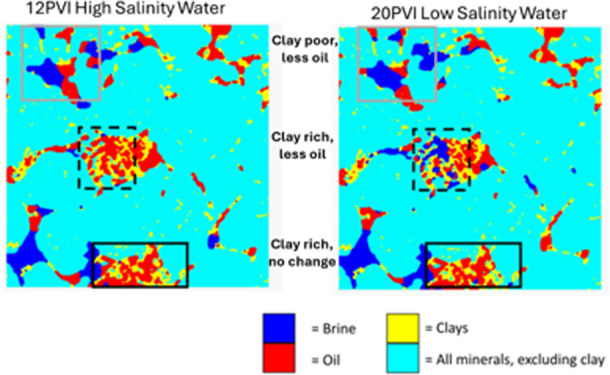

## Introduction

Low-salinity waterflooding is an enhanced oil recovery technique that has been observed to improve recovery by up to 14% in experiments over a range of scales.^1–4,4–12^ However, there have been many other studies that have observed little or no incremental oil recovery during low-salinity flooding.^13–17^ The disparities in experimental results have sparked a continuing drive to understand which systems will respond favorably to low-salinity flooding and which systems will not. However, as yet, no predictive model capable of identifying systems that will be responsive to low-salinity waterflooding has been developed.

Many authors suggested that a significant clay content is required for a favorable low-salinity response in sandstones; however, there is a growing body of work that contradicts this assertion. The first mention of clays in the context of low-salinity flooding was in 1967, with a pioneering paper by Bernard.^18^ General acceptance of the importance of clays emerged after^2^ concluded that the additional recovery during low-salinity flooding was not observed in clean sandstones. Subsequently, the vast majority of low-salinity waterflooding experiments were carried out using clay-rich sandstone samples.^1,4,6,19–24^ However, there is growing evidence that clays may not be vital for a favorable response to low-salinity flooding in sandstones. Favorable responses have been observed in clay-free sand-packs, clay-free sandstone samples, clay-free micromodels, and on quartz mineral surfaces.^25–32^ The response and importance of clays to low-salinity flooding is still an active area of scientific debate, and further observations across a range of scales are required to reconcile the contrasting observations within the field.

There is a lack of in situ pore-scale observations of the low-salinity response due to naturally heterogeneous clay distributions. In natural systems, the clays will form only 10% of the mineral content of a sandstone core and will be found most frequently in the smaller pores and coating fractions of the pore walls of the larger pores. Most of the core volume is thus associated with pores that have a lower clay content. The vast majority of experimental studies investigating the response of clays during low-salinity waterflooding have observed behavior on either the subpore scale or the core scale (where samples typically range from a few to tens of centimeters). On the subpore scale, clay-coated flow cells and micromodels have been used to observe wettability alteration and oil detachment from clay surfaces.^29,33–39^ The relevance of these experiments to natural systems is, however, unclear, as the flows are 2D, and the clay distribution within the pore networks is uniform. In core-scale experiments which do have a heterogeneous clay distribution, pressure drops and bulk saturation values are monitored, and the roles of clays and pore-scale processes, such as wettability alteration and fluid redistribution, are inferred but not directly observed.^1,4,19–23^ There remains a poor understanding of how a natural, heterogeneous clay distribution on the pore scale translates to production over core and reservoir scales.

Micro-CT X-ray imaging, which provides observations at micron resolution across millimeter-scale domains, has been used extensively to observe changes in wetting states and fluid distribution over pore and pore-network scales during low-salinity flooding,^40–46^ and Shabaninejad et al.^47^ have shown that it is also possible to segment clay from other mineral groups in X-ray μCT images of mineralogically heterogeneous sandstone samples. Furthermore, Garfi et al.^46^ used surface area fractional coverage to assess the wetting states of different mineral groups, including clays. However, these advances have not yet been applied to assess the pore-scale response of clays during low-salinity waterflooding in an oil-wet sample.

In this work, we make the first pore-scale in situ observations comparing the response of clay-rich and clay-poor pores during low-salinity flooding in oil-wetting sandstone samples. We present the results from tertiary, unsteady-state, low-salinity waterflood experiments on 3 sandstone samples of similar mineralogy but different pore structures. We analyze the resulting saturation changes, mineral-specific fluid-coated area fractions (developed by Garfi et al.^46^), and fluid distribution and present direct pore-scale observations of clay behavior to quantify the role of clays in the response to low-salinity waterflooding. This work provides unique insights into how clays respond to low-salinity flooding and the effect that this has on the distribution of oil and brine across the pore-network scale.

## Methods

### Rock Samples and Fluid Properties

We used three experimental data sets described by Andrews et al.^48^ These data sets consist of raw X-ray micro-CT images of tertiary low-salinity waterflooding experiments performed in Berea, Bunter, and Castlegate sandstone samples of altered wetting states. A brief description of the materials and methods for these experiments is included below.

### Materials

#### Rock Lithologies and Mineralogies

Outcrop samples from three different sandstone formations (Berea, Bunter, and Castlegate) were used in this study. They all have similar mineralogies (Table 1) and similar proportions of the different clay types (Table 2), except that only the Berea samples included a proportion of chlorites. In particular, the clay content of all samples includes between 20 and 30 wt % kaolinite. The properties of the cores used in this study are summarized in Table 3.

**Table 1:** Mineralogy by % Weight of Different Samples from the Same Outcrops as the Cores Used in This Research^a^

	quartz [%]	feldspar [%]	clays [%]	other [%]	
Berea 1	73.1	15.8	7.1	3.9	pyrite, calcite, ankerite, dolomite
Berea 2	70.3	15.1	7.4	7.2	calcite, pyrite, ankerite, dolomite
Bunter	76.8	12	9.8	1.4	hematite
Castlegate 1	94.6	1.5	3.7	0.2	halite
Castlegate 2	93.3	2.0	4.4	0.2	halite
Castlegate 3	91.9	2.6	4.3	0.8	jarosite, halite

a These were determined by XRD (Bunter and Castlegate) and XRF (Berea^45^). The minor minerals are listed in the order of decreasing amount. The samples were analyzed by different laboratories.

**Table 2:** % Clay by Weight Determined by XRD (Bunter and Castlegate) and XRF (Berea) from Different Samples of the Rocks Together with the % of the Different Clay Types Identified

	total clay [wt %]	smectite [%]	illite [%]	kaolinite [%]	chlorite [%]
Berea 1	7.1	32.6	23.9	29.6	14.1
Berea 2	7.4	25.7	32.4	21.6	20.3

		illite/smectite [%]	illite + mica [%]		
Bunter	9.8	7.2	67.5	24.6	0.0

		illite + illite/smectite [%]		
Castlegate 1	3.7	67.6	32.4	0.0
Castlegate 2	4.4	79.5	20.5	0.0
Castlegate 3	4.3	74.4	25.6	0.0

**Table 3:** Summary of Rock Properties for the Berea, Bunter, and Castlegate Samples Used in This Work^a^

	porosity	permeability [mD]	clay volume fraction	sample diameter [mm]	sample length [mm]
Berea	0.11	14	0.06	6	20
Bunter	0.15	142	0.06	6	15
Castlegate	0.20	495	0.08	5.5	15

a The porosity and clay fractions were determined directly from micro-CT images of the samples. The permeabilities were determined using pore-network modeling of flow through networks extracted directly from the images.^56^

The Berea core had a diameter of 6 mm and a length of 20 mm. Berea sandstone has been used extensively in petrophysical research.^45,49,50^ As shown in Table 1, it is predominantly made up of quartz (>70%) with smaller fractions of feldspar (<20%) and clays (<10%), as well as minor quantities of additional minerals such as pyrite, calcite, and ankerite.^45^ The range of porosity and permeability values for Berea sandstone is well-defined. Porosity is typically between 0.18 and 0.25, and the permeability values range from 45 to 1000 mD.^47,51,52^ The porosity and permeability of our sample are slightly lower than these typical values.

The Bunter core had a diameter of 6 mm and a length of 15 mm and was cut from a Triassic Sherwood Sandstone core supplied by the British Geological Survey. The mineralogy and petrophysical properties of this group vary dramatically; however, the sample from which our core was cut consisted of quartz and feldspar grains with nearly 10 wt % clay. Surveys across samples in this group have shown a wide range of porosity and permeability values, with values typically in the range of 0.05–0.4 and 10–600 mD, respectively.^53–55^

The Castlegate core had a diameter of 5.5 mm and a length of 15 mm and was cut from a block with porosity values in the range of 0.2–0.25, with a mean of 0.21, and permeability values in the range of 550–950 mD, with a mean of 750. The samples tested were 92–94 wt % quartz, 2 wt % feldspar, and 4 wt % clay minerals.

#### Fluid Properties

Two distinct brines were used for all of the experiments. The first, referred to as high-salinity brine, was used as the formation brine for each sample and for the secondary waterflood in each experiment. The second, referred to as low-salinity brine, was used for subsequent tertiary waterfloods. Both brine recipes are shown in Table 4. The low-salinity brine is simply the high-salinity brine recipe diluted by a factor of 69. This resulted in a relatively low TDS for the low-salinity brine, which has been seen to result in clay destabilization and mobilization.

**Table 4:** Brine Recipes for Both the High- and Low-Salinity Brines Used in This Study^a^

	dissolved salts [mg/L]	
salts	high-salinity brine	low-salinity brine
CaCl_2_·2H_2_O	13,205	190
MgCl_2_·6H_2_O	2008	29
KCl	744	11
NaCl	57,884	834
TDS	73,841	1064

a Note that the low-salinity brine is simply the high-salinity brine diluted by a factor of 69.

The same oil was used for all three experiments: a degassed crude oil with a density of 0.87 kg/m^3^ and a viscosity of 13 mPa s at 70 °C. It has a total acid number of 0.01 mgKOH/g and a total basic nitrogen of 2000 mg/kg. A crude oil was chosen to maximize the wetting alteration during aging, as previous work^6^ has suggested that a significant resin and asphaltene content is needed for a positive low-salinity effect. These compounds are believed to be the most active in causing reservoirs to become mixed or oil-wet.^57^ Multicomponent ion exchange then results in these compounds being released from the pore wall, changing the pore wettability. Analysis of the saturates, aromatics, resins, and asphaltenes gave Sat = 22.00 wt %, Aro = 41.00 wt %, Res = 20 wt %, and Asp = 17 wt %.

The oil was doped with 20 wt*%* iododecane. This dopant concentration represents a trade-off between replicating a realistic reservoir system and the ability to reliably distinguish between phases in the X-ray images. We added the dopant to the oil instead of the brines to avoid increasing the salinity of the low-salinity brine. There remains some uncertainty around the effect of iododecane on interfacial properties, rock wettability, and other interactions. Pan and Trusler^58^ reported that the interfacial tension between iododecane and water was 4.5 mN/m lower than the interfacial tension between water and decane. This would suggest that the oil–water IFT could be modified in these experiments. As noted by Pairoys et al.,^59^ the impact of iododecane on other properties is yet to be established.

### Flow Experiments

Each sample was obtained from an outcrop that had never been exposed to crude oil. This meant that they were initially water-wet and needed to be aged to achieve the initial mixed to oil-wet state, typical of an oil reservoir.

First, each core was mounted in a carbon fiber Hassler-type flow cell, which was used to keep them under pressure with a confining fluid (water). The samples were placed in a Viton sleeve and connected to a hydraulic circuit with two steel end pieces. The cell was then placed into an FEI HeliScan X-ray μ-CT instrument so that images could be taken at each stage of the experiment. For all flow steps, a confining pressure of 50 bar was maintained within the core holder. This compressed the Viton sleeve around the core, thereby preventing the fluid from bypassing the sample. An inlet pressure of 30 bar was maintained at all times. The experiments were carried out at ambient temperature of approximately 25 °C.

Each flow experiment followed the same basic workflow, as summarized in Figure 1. High-salinity brine was first pumped through each sample at 0.2 mL/min for 30 min to saturate the sample fully. Undoped crude oil was then pumped through the samples for 10 pore volumes. The wetting state of the cores was then altered by exposure to the crude oil. This was achieved by submerging the samples in a sealed beaker of undoped crude oil for 4 weeks at 80 °C. Next, high-salinity brine was pumped through the samples for a total of 12 pore volumes. Four pore volumes of low-salinity brine were then injected, followed by a final 16 pore volumes of low-salinity brine injection, making a total of 20 pore volumes of low-salinity flooding.

**Figure 1:**
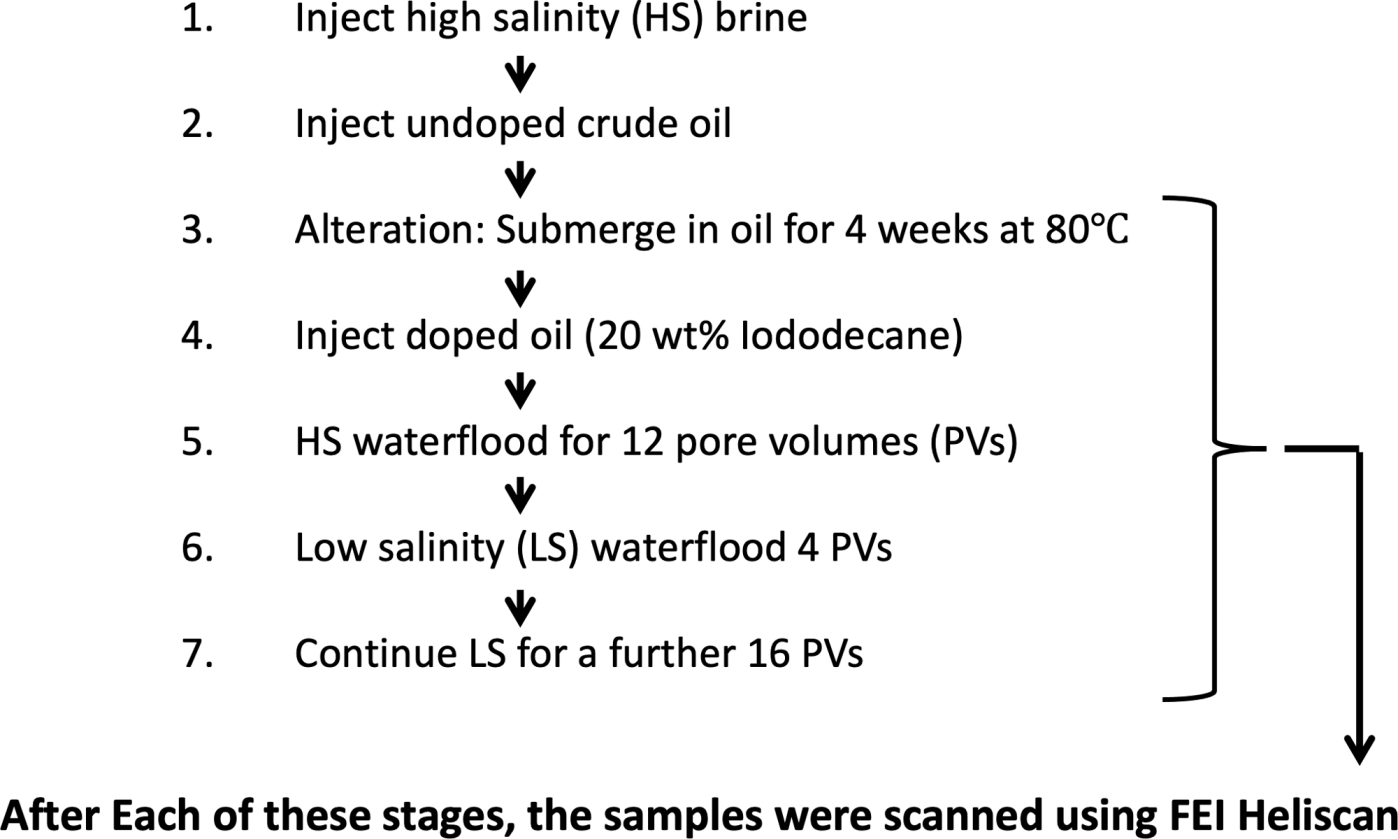
Overview of the experimental workflow for all samples. A more detailed description of the workflow can be found in the text.

The flow rates were chosen to ensure a capillary number close to that typical of field-scale displacements. For the Bunter and Castlegate samples, this corresponded to a flow rate of 0.015 mL/min at an injection pressure of 30 bar, which gives an approximate capillary number for the waterfloods of 3.6 × 10^–7^ and 3 × 10^–7^, respectively. For the Berea sample, we could not achieve this flow rate. Instead, we maintained a constant pressure drop of 25 bar for these flow steps. This resulted in an average flow rate of 0.001 mL/min, giving an approximate capillary number for the waterfloods of 2 × 10^–8^. Evidently, the aging process in the Berea sandstone led to a reduction in permeability, possibly because of the precipitation of asphaltenes. We saw no evidence of capillary end effect in our experiments at these capillary numbers (see Figure 5) although it is possible that the entire core in each case is within the capillary end effect zone due to their small length.

### Image Analysis Workflow

In each experiment, after every flow step, the samples were scanned using an FEI Heliscan X-ray μ-CT machine. A voxel resolution of 2.4 μm was achieved for each sample. A region of interest was then extracted from all scans. For the Berea, the region of interest was 1500 × 1500 × 3500 voxels (3.6 × 3.6 × 8.4 mm); for the Bunter, it was 1500 × 1500 × 3000 voxels (3.5 × 3.5 × 7.0 mm); and for the Castlegate, it was 1425 × 1425 × 2375 voxels (3.3× 3.3 × 5.5 mm). The signal-to-noise ratio of all images was increased using a nonlocal means filter.^60^

Figure 2 shows the image analysis workflow for the Berea sample. An identical image analysis workflow was followed for the Bunter and Castlegate samples. First, the undoped scans (Figure 2a) were segmented into 3 phases—the pore space and two mineral groups, namely, 1) clay minerals and 2) nonclay minerals—using a watershed segmentation.^61^ The segmented image was then used to mask all subsequent scans (Figure 2c). After each image was masked, just the fluid phases remained (Figure 2d). Thresholding was then used to segment the brine and oil, where the threshold value was determined by the histogram of gray values. The segmented fluid phases were then combined with clay segmentation. Lastly, an erosion/dilation tool was used to remove any erroneous layers with a thickness of one voxel from the mineral surfaces (Figure 2e).

**Figure 2:**
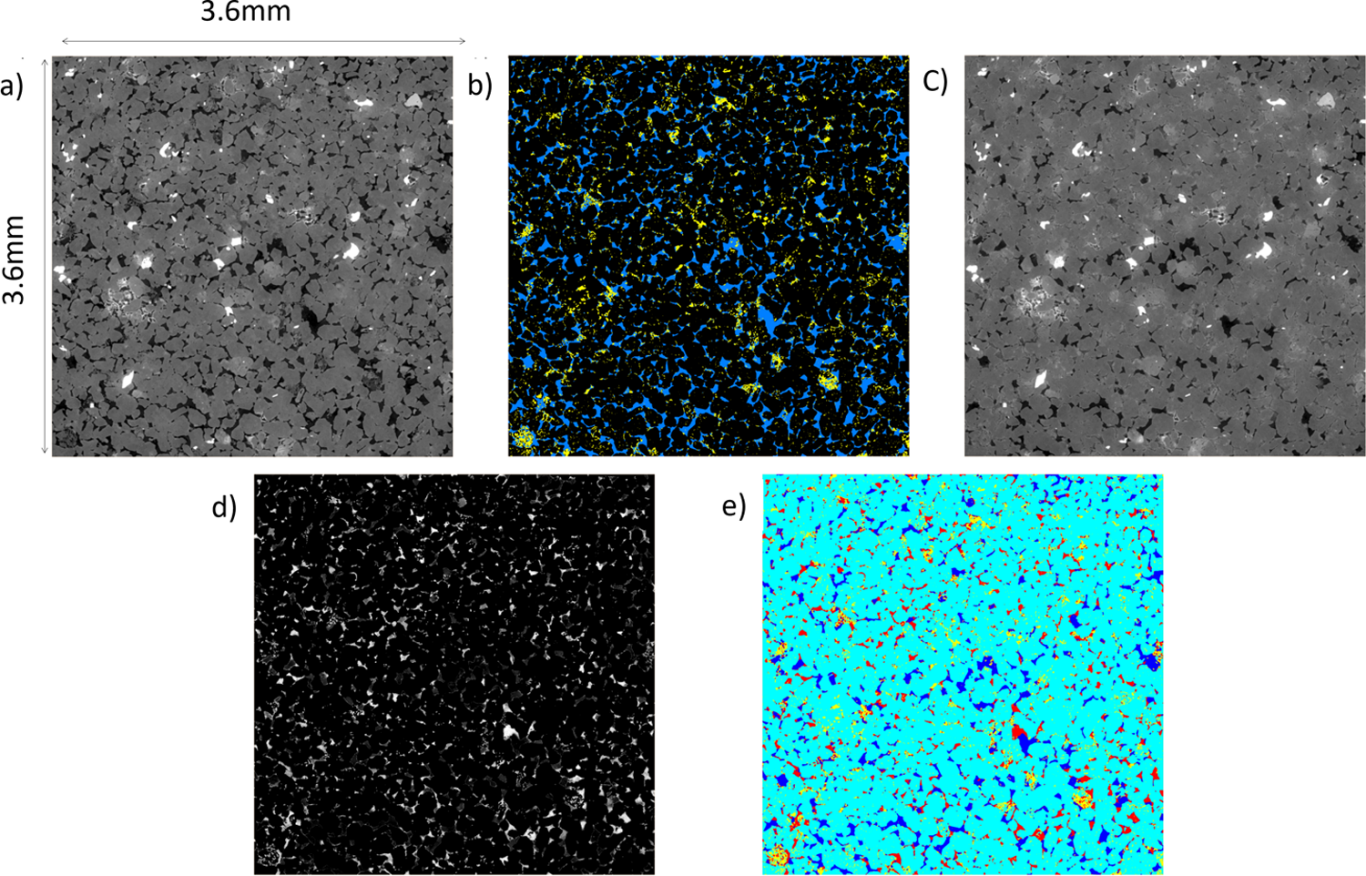
Overview of the image processing workflow. (a) Undoped scan (filtered). (b) Segmented undoped scan (blue = pore space, black = rock, yellow = clay), which is then used as a mask for the doped scans. (c) Scan taken after 20 pore volumes of low-salinity waterflooding (filtered). (d) As in panel (c) but masked using panel (b), leaving just the fluid phases (light = oil, dark = brine). (e) Segmented LSWF 20PV scan (light blue = rock, dark blue = brine, red = oil, yellow = clay).

### Calculating Saturations and Mineral-Specific Surface Area Coverage, Pore by Pore

The fluid saturation across the region of interest was estimated using the segmented images, where the water saturation, *S*_w_, was averaged across each of the horizontal slices (perpendicular to the flooding direction) in each sample. The values were then stacked to produce a profile of saturation values along the length of the region of interest, as well as averaged to give the total water saturation, *S*_w_, in the region of interest.

We used a workflow developed by Garfi et al.^46^ to measure the fractional coverage of each pore’s walls by minerals, together with the fractional coverage of those minerals by oil and water. This allowed mineral-specific wettability to be analyzed and for the behavior of clay minerals to be isolated. We first split each sample into equal subvolumes. For the Berea, the region of interest was split into 63 equal subvolumes of 500 voxels per side; for the Bunter, the region of interest was split into 54 equal subvolumes of 500 voxels per side; and for the Castlegate, the region of interest was split into 45 equal subvolumes of 475 voxels per side. For each subvolume, we identified four interface groups, that is, two mineral groups (clay minerals and nonclay minerals) and two fluid phases (brine and oil). We compared fluid surface coverage of the two mineral groups by recording the fraction of the total area of that mineral group in contact with a fluid:

$$a_{ij}=\frac{A_{ij}}{\sum_{i}A_{ij}}$$ 1

where *A*_*ij*_ is the measured surface area per unit of pore volume shared by fluid *i* with solid *j*, respectively. We measured the oil–clay interfacial area as a fraction of the total fluid–clay interfacial area and the oil–mineral interfacial area for nonclay minerals as a fraction of the total fluid–mineral interfacial area for nonclay minerals and presented this value as a function of oil saturation, *S*_o_, for each subvolume.

We evaluated the pore sizes occupied by fluid phases using a pore-network abstraction of the pore space, in a workflow first introduced by Bultreys et al.^56^ This involves using a maximal ball network extraction code to extract a network of nodes representing pores and links representing throats from the segmented undoped scan of each sample. Inscribed spheres are fitted to each pore, where the diameter of said spheres represents the pore diameters.^62,63^ These spheres were then mapped onto the segmented images of each flow step. The pore occupancy of brine and oil was then quantified by using segmented images to determine the phase occupying each sphere.

For each identified pore, we calculated the fraction of the pore surface that is coated by clay. We then grouped pores into two groups: (1) clay-rich pores, where >50% of pore surface is coated by clay minerals, and (2) clay-poor pores, where <50% of pore surface is coated by clay minerals. We then calculated the pore radius distribution of brine-occupied clay-rich and clay-poor pores.

Andrews et al.^44^ used the methods described above to calculate the saturation values, fluid-coated area fraction values, and pore occupancy for a water-wet Berea sandstone sample after secondary high-salinity waterflooding and two tertiary low-salinity waterfloods. *S*_w_ values of 0.260, 0.256, and 0.254 were recorded after high-salinity waterflooding, four pore volumes of low-salinity waterflooding, and 20 pore volumes of low-salinity waterflooding, respectively. This gives a mean value of 0.257, and a standard deviation from the mean of 0.00125. There were similarly small changes in area fraction and pore occupancy measurements between the three waterfloods, indicating very little fluid displacement and redistribution during the low-salinity waterfloods. The average *A*_fo_ values for all subvolumes were calculated to be 0.641, 0.646, and 0.646 after high-salinity waterflooding, four pore volumes of low-salinity waterflooding, and 20 pore volumes of low-salinity waterflooding, respectively. This gives a mean value of 0.644 and a standard deviation from the mean of 0.00118. These small changes are consistent with larger-scale core floods and pore-network modeling of two-phase flows in water-wet samples. They also confirm that the image analysis workflow is robust and repeatable.

## Results and Discussion

### Clay Distribution

As shown in Tables 1 and 3 as well as Figure 3, all 3 outcrop samples have a significant clay fraction, with clay weight fractions of 7, 10, and 4% determined by XRD/XRF for different Berea, Bunter, and Castlegate samples compared with volume fractions of 6, 6, and 8% determined directly from the micro-CT images of the cores. Some of these differences may be due to natural variability; however, there will also be differences due to the different measurement approaches. The micro-CT identifies clays that coat the pore surfaces and has a limited spatial resolution of 2 μm, whereas the XRD/XRF measures all the clays in a sample, whether on the surface or within the sand grains. This has previously been investigated in depth by Lai et al.^64^

**Figure 3:**
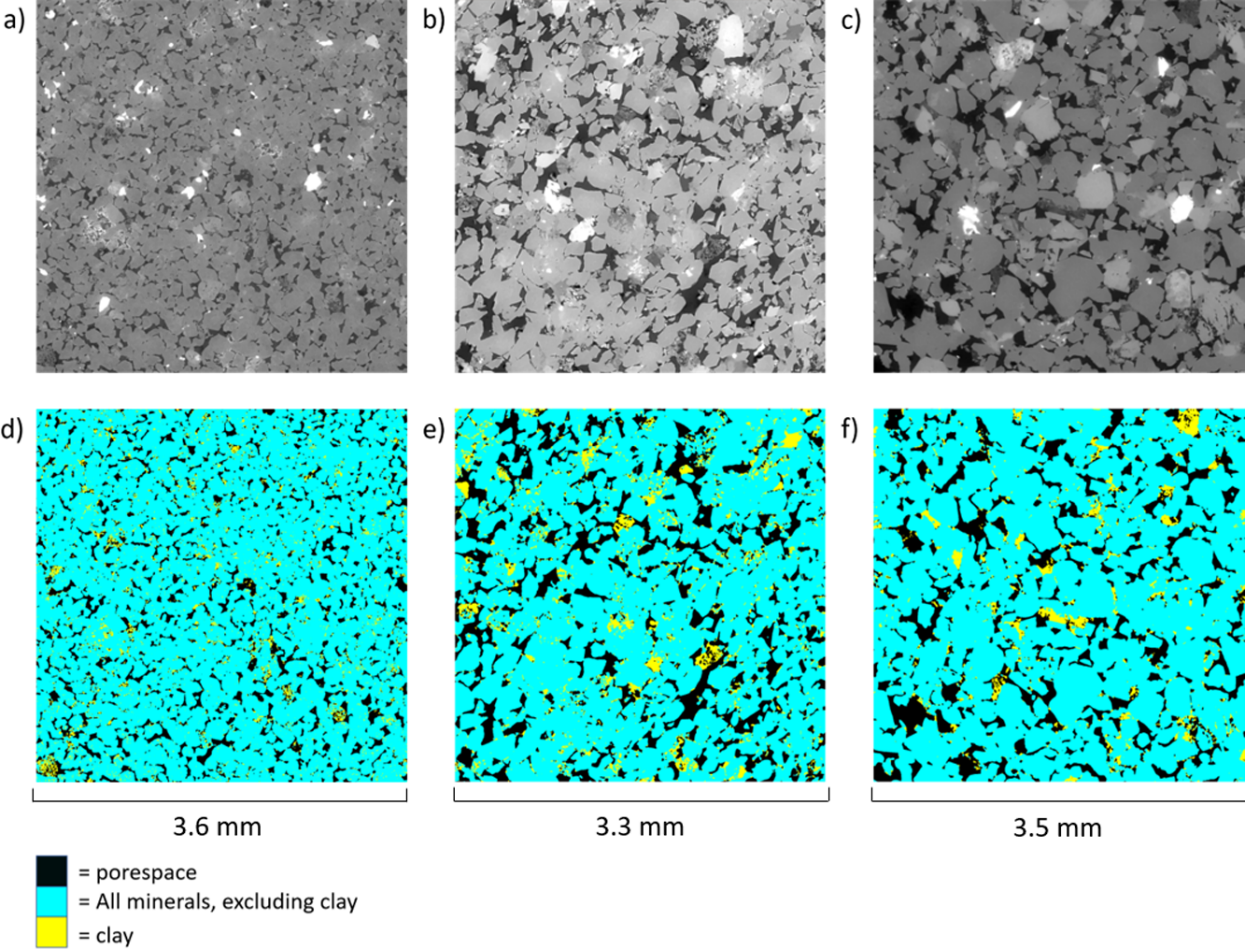
Slice of the X-ray micro-CT image of the (a) Berea sample, (b) Castlegate sample, and (c) Bunter sample before the injection of doped oil. In each case, the darkest color represents the pore space. In each case, the bright spots represent the small quantities of highly attenuating metallic minerals. The corresponding slices for the segmented images of the (d) Berea sample, (e) Castlegate sample, and (f) Bunter sample are also shown, where black represents the pore space, yellow represents clay minerals, and blue represents nonclay minerals.

Despite the similar clay fractions in the different samples, the percentage of the pore volume consisting of clay-rich pores differs significantly between each sample. Clay-rich pores make up 7% of the total pore volume in the Berea sample, 14% of the pore volume in the Bunter sample, and 4% of the total pore volume in the Castlegate sample. Figure 4 shows the fraction of pores in each sample that are clay-rich (>50% clay coverage) for different pore radii. In all samples, smaller pores are preferentially clay-rich, where >50% of pores with a radius of <7 μm are clay-rich in each sample. In all samples, large pores are preferentially clay-poor, and this is most pronounced in the Berea and Castlegate samples, where <5% of pores with a radius of >15 μm are clay-rich. In the Bunter sample, the smallest pores are also more likely to be clay-rich, with >60% of the smallest pores being clay-rich. In contrast with the other two samples, a significant fraction, specifically 15%*,* of pores >35 μm radius are clay-rich in the Bunter sample.

**Figure 4:**
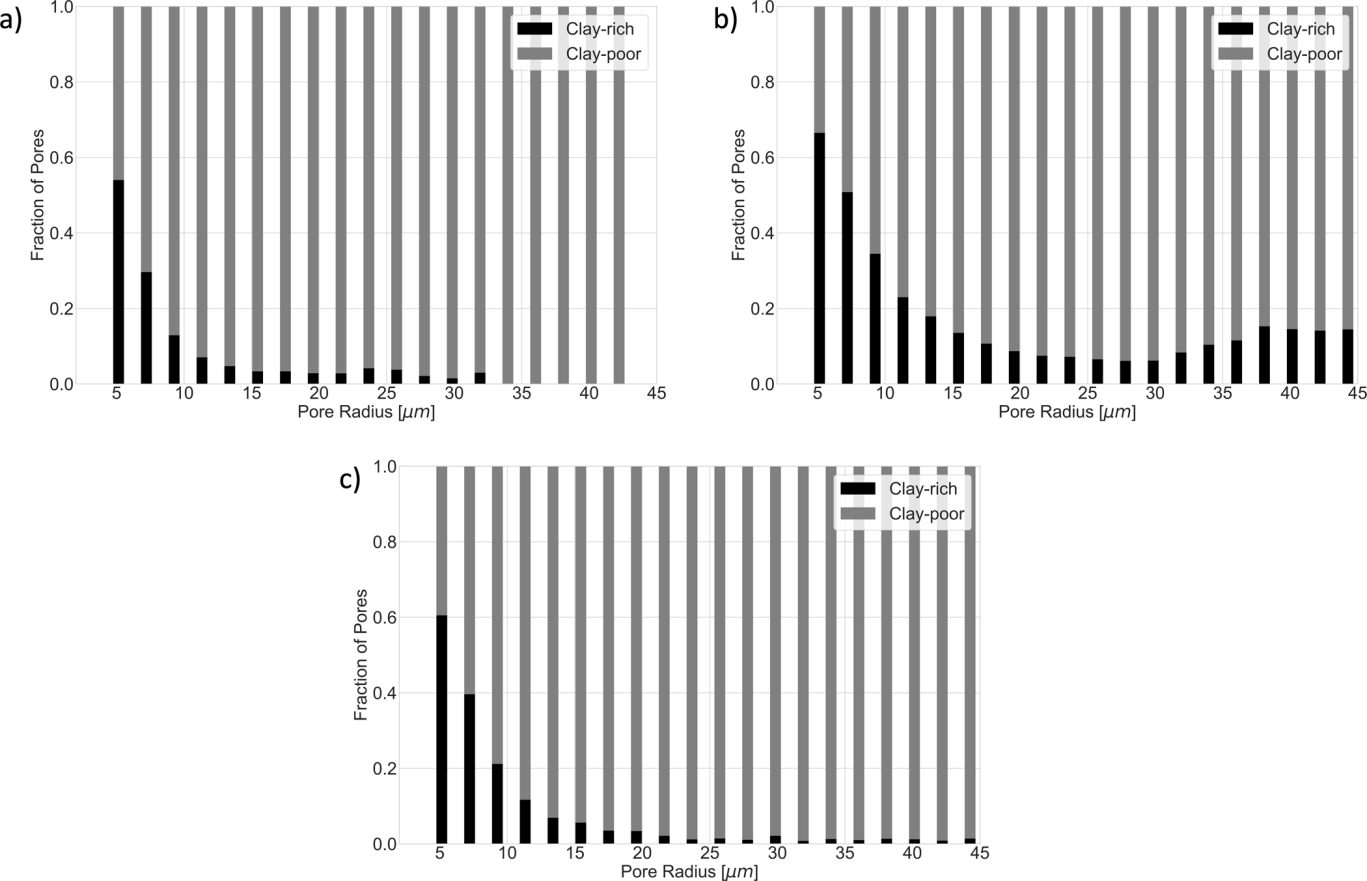
Fraction of clay-rich (> 50%clay coverage) pores in different radii bins for (a) Berea, (b) Bunter, (c) Castlegate samples. In each case, the smaller pores are preferentially clay-rich. In the Berea and Castlegate samples, there are very few clay-rich large pores (>15 μm). In contrast, in the Bunter sample, a larger fraction of larger pores are clay-rich, approximately 15% of pores (>35 μm) are clay-rich. Clay-rich pores make up 7% of total pore volume in the Berea sample, 14% of pore volume in the Bunter sample, and 4% of total pore volume in the Castlegate sample.

### Saturation Distribution

Figure 5 shows the saturation profiles for each of the three samples after each waterflood step. Significant additional production of oil was observed after 20 pore volumes of low-salinity waterflooding in the Berea and Bunter samples (Figure 5a,c, respectively), with additional recoveries of three and four percentage points, respectively (Table 5). In the Castlegate sample (Figure 5b, 1 percentage point of additional recovery was observed after low-salinity waterflooding. The local changes in *S*_w_ values after 20 pore volumes of low-salinity flooding in the Castlegate sample are significantly smaller than the changes observed in both the Berea and Bunter samples.

**Figure 5:**
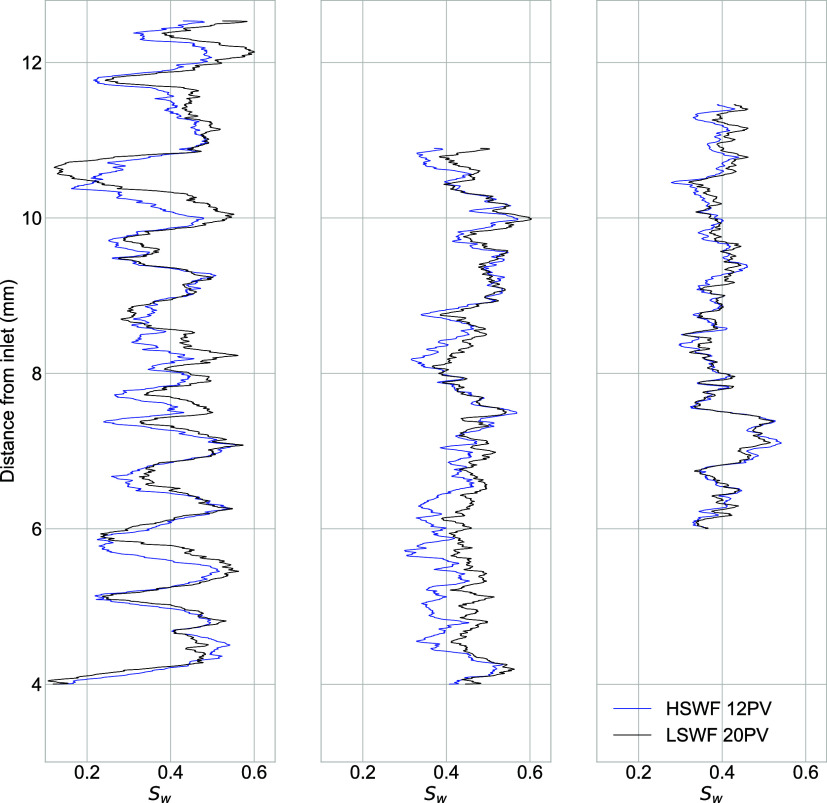
Profile of water saturation, *S*_w_, averaged perpendicular to the flooding direction across the region of interest after 12 pore volumes of high- salinity waterflooding (HSWF 12PV) and 20 pore volumes of low-salinity waterflooding (LSWF 20PV) for the (a) Berea sample, (b) Bunter sample, and (c) Castlegate sample. There were significant local changes in *S*_w_ following LSWF 20PV in the Berea and Bunter samples and smaller changes in the Castlegate sample. These resulted in additional recoveries of 3, 4, and 1 percentage points in the Berea, Bunter, and Castlegate samples, respectively.

**Table 5:** Average Water Saturation, *S*_w_, in the Region of Interest for All Samples after 12 Pore Volumes of High-Salinity Flooding (HSWF) and 20 Pore Volumes of Low-Salinity Waterflooding (LSWF 20PV)^a^

	Berea *S*_w_	Bunter *S*_w_	Castlegate *S*_w_
HSWF 12PV	0.382	0.429	0.389
LSWF 20PV	0.413	0.467	0.400

a In all cases, there was a very high initial oil saturation with *S*_w_ < 0.05 prior to waterflooding.

The observed recovery values in the Berea and Bunter samples are similar to the results from several studies carried out on sandstones under similar conditions: Lebedeva and Fogden^65^ observed an additional recovery of 7 percentage points during low-salinity flooding of a kaolinite-coated sandpack; Chen et al.^43^ reported a recovery of 5 percentage points after tertiary low-salinity flooding in the Berea Sandstone; and Shabaninejad et al.^47^ observed a recovery of 3 percentage points in a tertiary low-salinity waterflood in the Berea sandstone sample.

### Mineral-Specific Surface Area Fractional Coverage

Figure 6 shows mineral-specific surface area fractional coverage values for all three samples after 12 pore volumes of high-salinity waterflooding and then after 20 pore volumes of low-salinity waterflooding. In each sample, the *A*_fo_(*S*_o_) values for clay are distinct from those of the other minerals for all flow steps. For a given *S*_o_, we observe that the fraction of clay surfaces coated by oil is significantly higher than the fraction of nonclay mineral surfaces coated by oil. These results show that clays are more oil-wetting than nonclay minerals in all three samples. This is a finding consistent with observations by Garfi et al.,^46^ who reported higher fractional oil coverage on clays than on quartz and feldspar within a Berea sample of altered wettability, and Tabrizy et al.,^66^ who found powdered kaolinite to be more prone to wettability alteration during exposure to crude oil than quartz and feldspar.

**Figure 6:**
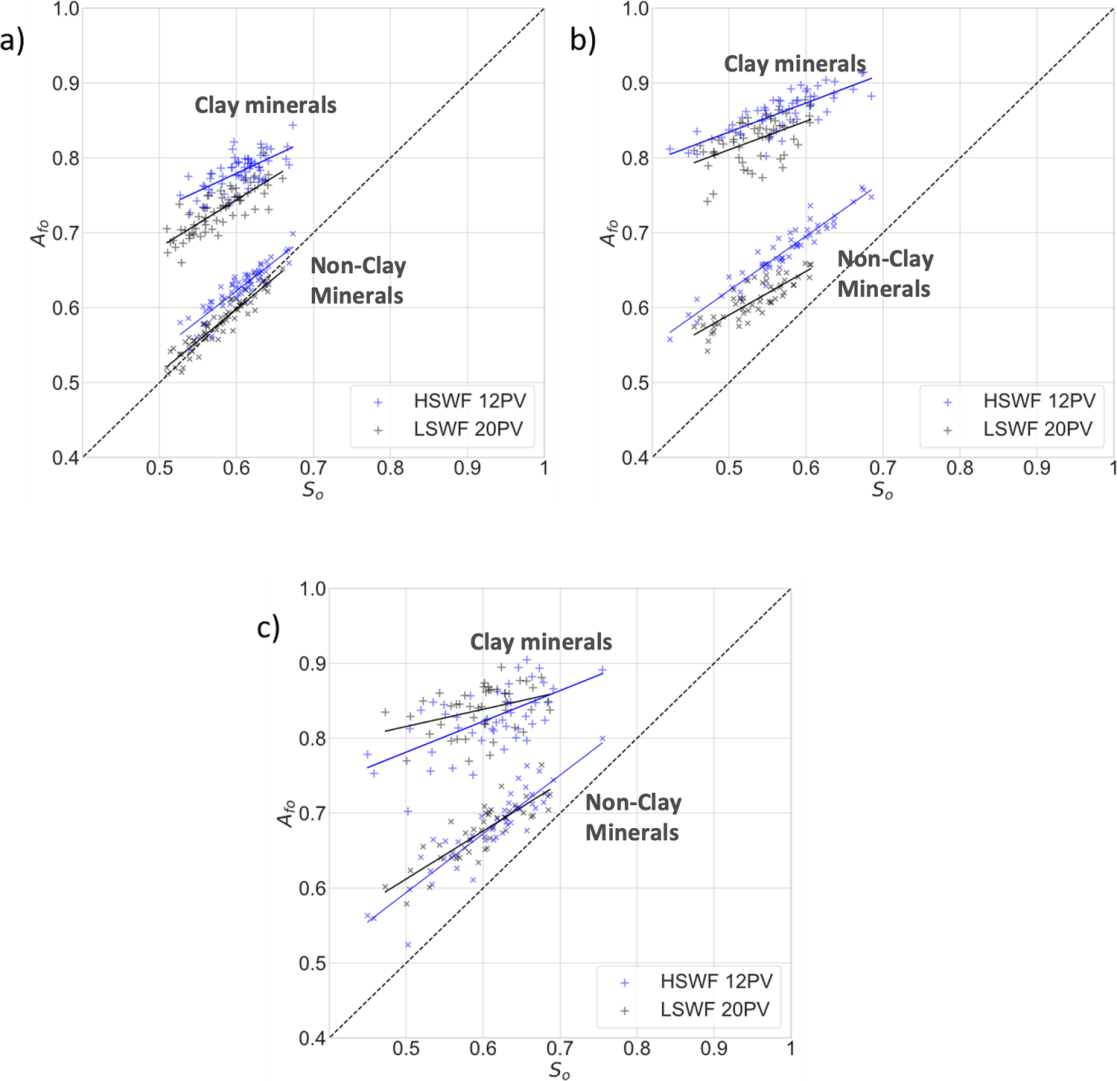
Mineral-specific oil-coated area fractions, imaged after high-salinity waterflooding (HSWF) and 20 pore volumes of low-salinity flooding (LSWF 20PV) for (a) Berea, (b) Castlegate, and (c) Bunter. For each sample, *A*_fo_(*S*_o_) values are plotted for both clay surfaces and the surfaces of nonclay minerals. In each case, *A*_fo_(*S*_o_) values are greater for clays than nonclay minerals. There is a significant decrease in *A*_fo_(*S*_o_) values for both mineral groups after LSWF 20PV. This change was noticeable after 4 pore volumes of low-salinity water injection after which time there was no significant change in *A*_fo_(*S*_o_) values in either the Berea or Bunter samples, although there is a significant change to lower *S*_o_ values. In the Castlegate sample, there is no clear shift in *A*_fo_(*S*_o_) values for either mineral group after either LSWF 4PV or LSWF 20PV.

We observe that the *A*_fo_(*S*_o_) values for both mineral groups are lower in the Berea sample than for the Bunter and Castlegate samples after either waterflood. This suggests that both mineral groups in the Berea sample are initially less oil-wet than those in the Bunter and Castlegate samples. In the Berea sample, after 20 pore volumes of tertiary low-salinity flooding, there is a shift to lower *A*_fo_(*S*_o_) values in both mineral groups, so that for a given *S*_o_, there is a lower fraction of oil coating the mineral surfaces. This behavior is most significant for clay minerals. This indicates a shift to more water-wet conditions in both mineral groups, with a more pronounced shift in clay minerals. In the Bunter sandstone, there is a significant shift to lower *A*_fo_(*S*_o_) values in both mineral groups after 20 pore volumes of tertiary low-salinity flooding (LSWF 20PV). This wettability alteration in the Bunter sample is greater on nonclay minerals than on clay minerals. In the Castlegate sample, there is no significant shift in *A*_fo_(*S*_o_) values in either mineral group after 20 pore volumes of low-salinity waterflooding. This indicates no pervasive and systematic wettability alteration during low-salinity waterflooding within the Castlegate sample.

Observations of wettability alteration during low-salinity flooding in the Berea and Bunter sandstone samples are broadly consistent with a wealth of observations of wettability alteration during low-salinity waterflooding across a range of scales.^11,33,34,36,37,43,47,67,68^ More specifically, observations of wettability alteration on clay minerals in the Berea sample, and to a lesser extent, the Bunter sample, are consistent with several studies using flow cells to observe oil detachment on clay basal planes,^33,34^ the detachment of oil droplets from clays deposited on a glass substrate,^36,37^ and in situ observations of oil detachment from clays in the presence of low-salinity brine using atomic force microscopy.^67^ Observations of wettability alteration on nonclay minerals in the Berea and Bunter sandstone samples agree with a growing body of research, suggesting that significant wettability alteration can occur on quartz and feldspar grains during low-salinity flooding.^29–32,47^

### Pore Occupancy

To understand the effect of any wetting alteration on fluid distribution throughout each sample and further understand the role of mineralogy in the low-salinity response, we next assessed pore occupancy. Andrews et al.^48^ presented pore occupancy analysis for all pores within the region of interest of each sample. Here, we extend that analysis by observing mineral-specific pore occupancy and analyzing the pore occupancy of clay-rich pores (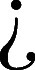
50% clay coverage) and clay-poor pores (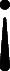
50% clay coverage) separately. This allows for the role of clays in pore occupancy changes to be investigated while recognizing that the micro-CT cannot resolve all the clay particles.

We observe that clay-poor pores account for the vast majority of oil displacement events following low-salinity waterflooding in each sample. Figure 7 shows the pore occupancy of clay-poor and clay-rich pores in each sample for a range of pore radii as a fraction of total pore volume. There is a significant increase in brine saturation in the smallest clay-poor pores for each sample (pore radius <18 μm in the Berea sample, <30 μm in the Bunter sample, and <28 μm in the Castlegate sample). This effect is the largest in the Berea and Bunter samples. Observations of pore occupancy changes in small and medium pores during low-salinity flooding are consistent with a range of studies. Experimental studies across both sandstones and carbonates^41,68,69^ and modeling studies^70^ observe that brine saturation increases in the smallest pores during low-salinity waterflooding. This effect is thought to be the result of a wettability alteration to more water-wetting conditions. The alteration allows water to more easily enter smaller pores and throats because of the support of capillary forces.^41,70^

**Figure 7:**
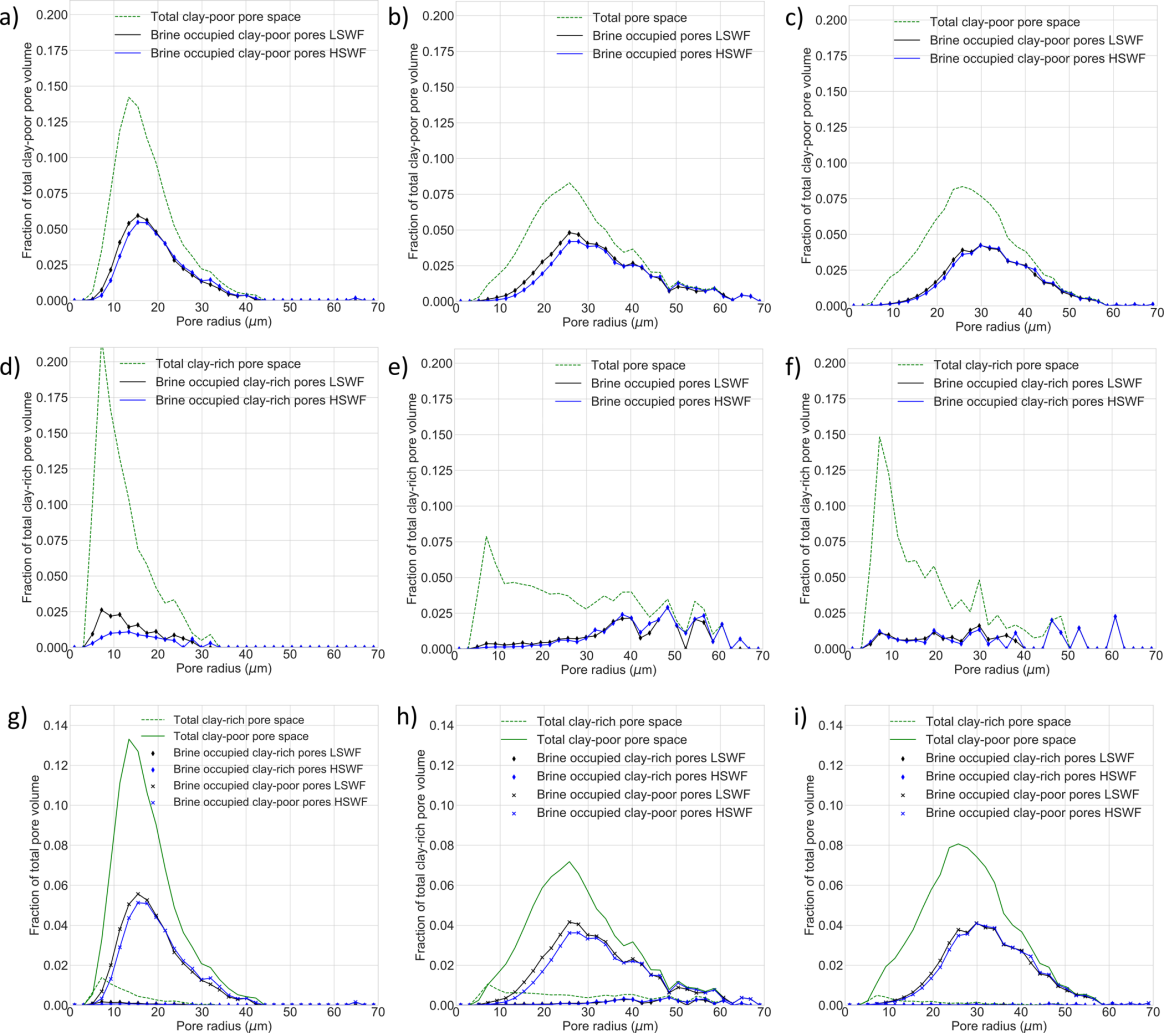
Size distribution for all brine-saturated clay-poor pores (bodies and throats) in the (a) Berea, (b) Bunter, and (c) Castlegate samples and the size distribution for all brine-saturated clay-rich pores (bodies and throats) in the (d) Berea, (e) Bunter, and (f) Castlegate samples after high-salinity waterflooding (HSWF) and low-salinity flooding (LSWF). A comparison of the size distribution for all brine-saturated clay-poor and clay-rich pores is presented for (g) Berea, (h) Bunter, and (i) C astlegate. In all samples, there is an increase in brine volume in the small and medium clay-poor pores after LSWF, and this is most significant in the Berea and Bunter samples. There is also an increase in brine volume in the small and medium clay-rich pores in the Berea and Bunter samples. In all cases, clay-poor pores are larger and more abundant than clay-rich pores and thus account for the majority of total pore occupancy changes by volume.

For clay-rich pores in the Berea sample, there is a large increase in brine saturation in small pores, with the fraction of pores of radius <12 μm occupied by brine more than doubling during low-salinity flooding. In contrast, in the Bunter and Castlegate samples, there are far smaller changes in pore occupancy in clay-rich pores across all pore radii. In both samples, the relative pore occupancy changes in clay-poor pores are significantly greater than those in clay-rich pores. When coupled with the fact that clay-rich pores account for only a small fraction of total pore space in each sample, it is clear that oil production from clay-rich pores in the Bunter and Castlegate samples is very small. This can be seen in Figure 7g–i, which show the relative importance of pore occupancy changes in pores of each mineral group. We can analyze this effect quantitatively (Figure 8). In the Berea sample, clay-rich pores make up 7% of pore volume and account for 15% of the total additional recovery during low-salinity flooding. In the Bunter sample, clay-rich pores make up 14% of total pore volume and account for 5% of the total additional recovery during low-salinity flooding. In the Castlegate sample, clay-rich pores make up 4% of total pore volume and account for 3% of the total additional recovery during low-salinity flooding. Even in the Berea sample, where there is a pronounced wettability alteration on clay minerals and significant pore occupancy changes in clay-rich pores, the majority of oil production occurs from clay-poor regions of the pore space.

**Figure 8:**
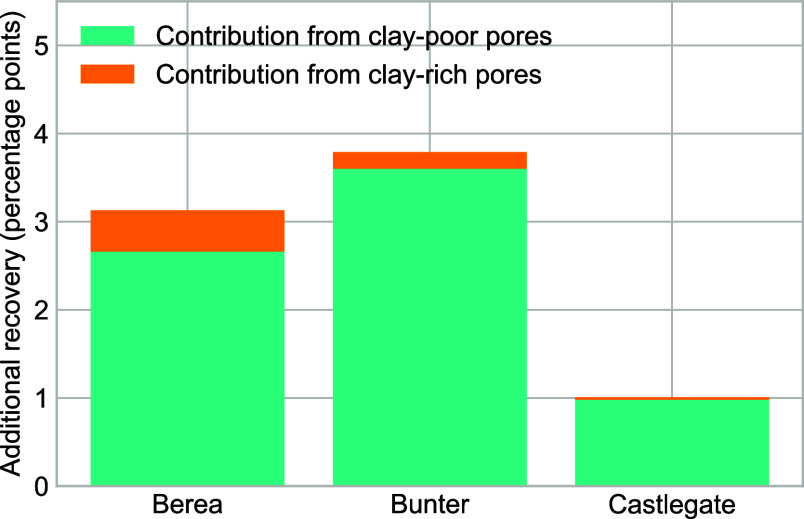
Bar chart showing the contribution of clay-rich and clay-poor pores to additional recovery during low-salinity flooding in each sample. Clay-rich pores account for 15% of the additional production in the Berea sample, 5% of the additional production in the Bunter sample, and 3% of the additional production in the Castlegate sample. Therefore, in all samples, clay-poor pores account for the majority of oil production.

In Figure 7, we observe that brine saturation in clay-rich pores is low for all samples. The *S*_o_ values for all clay-rich and clay-poor pores are given in Table 6. For all samples, clay-rich pores have significantly higher *S*_o_ values than clay-poor pores after both high-salinity flooding and low-salinity flooding. This effect is most prominent in the Berea and Castlegate samples, with *S*_o_ in clay-rich pores >20 percentage points higher than in clay-poor pores after both high- and low-salinity flooding for both samples. In the Bunter sample, probably because a larger fraction of large pores are clay-rich (Figure 4), *S*_o_ values in clay-rich pores are lower than those in the other two samples. However, *S*_o_ values are still significantly higher in the clay-rich pores than in the clay-poor pores, with a difference of 16 and 18 percentage points after high- and low-salinity flooding, respectively. The higher *S*_o_ values in clay-rich pores in all samples are to be expected, as we have shown that clays are preferentially present in the smallest pores (Figure 4) and are more oil-wetting than nonclay minerals in all samples (Figure 6). These effects combine to make brine invasion in clay-rich pores very unfavorable, as, in an oil-wet system, brine invasion preferentially occurs in larger, less oil-wetting pores.^71^

**Table 6:** *S*_o_ Values for Clay-Poor Pores (<50% Clay Coverage), Clay-Rich Pores (>50% Clay Coverage), and Very Clay-Rich Pores (>90% Clay Coverage) for All Samples after Both High-Salinity Waterflooding and Low-Salinity Waterflooding^a^

	clay-poor pores		clay-rich pores		very clay-rich pores	
	*S*_o_ HSWF	*S*_o_ LSWF	*S*_o_ HSWF	*S*_o_ LSWF	*S*_o_ HSWF	*S*_o_ LSWF
Berea	0.60	0.57	0.89	0.82	0.98	0.93
Bunter	0.55	0.50	0.73	0.72	0.96	0.96
Castlegate	0.57	0.56	0.80	0.79	0.97	0.96

a In all cases, there are significantly higher *S*_o_ values in clay-rich pores than clay-poor pores.

The high *S*_o_ in clay-rich regions is even more prominent when only considering very clay-rich pores (pores with >90% clay coverage). Very clay-rich pores are almost entirely oil-saturated after high-salinity flooding for all samples, with *S*_o_ values of 0.98, 0.96, and 0.97 in the Berea, Bunter, and Castlegate samples, respectively (Table 6). After low-salinity waterflooding, there is no significant change in *S*_o_ in very clay-rich pores in either the Castlegate or Bunter sample, so that very clay-rich pores remain almost entirely oil-saturated. In contrast, there is a 5 percentage point decrease in *S*_o_ in the very clay-rich Berea pores during low-salinity waterflooding. In the Bunter and Castlegate samples, very clay-rich pores remain almost entirely oil-saturated throughout the duration of the experiment. These observations are further illustrated in Figures 9, 10, and 11, which show direct observations of fluid occupancy in clay aggregates within subvolumes of the Berea, Bunter, and Castlegate samples, respectively.

**Figure 9:**
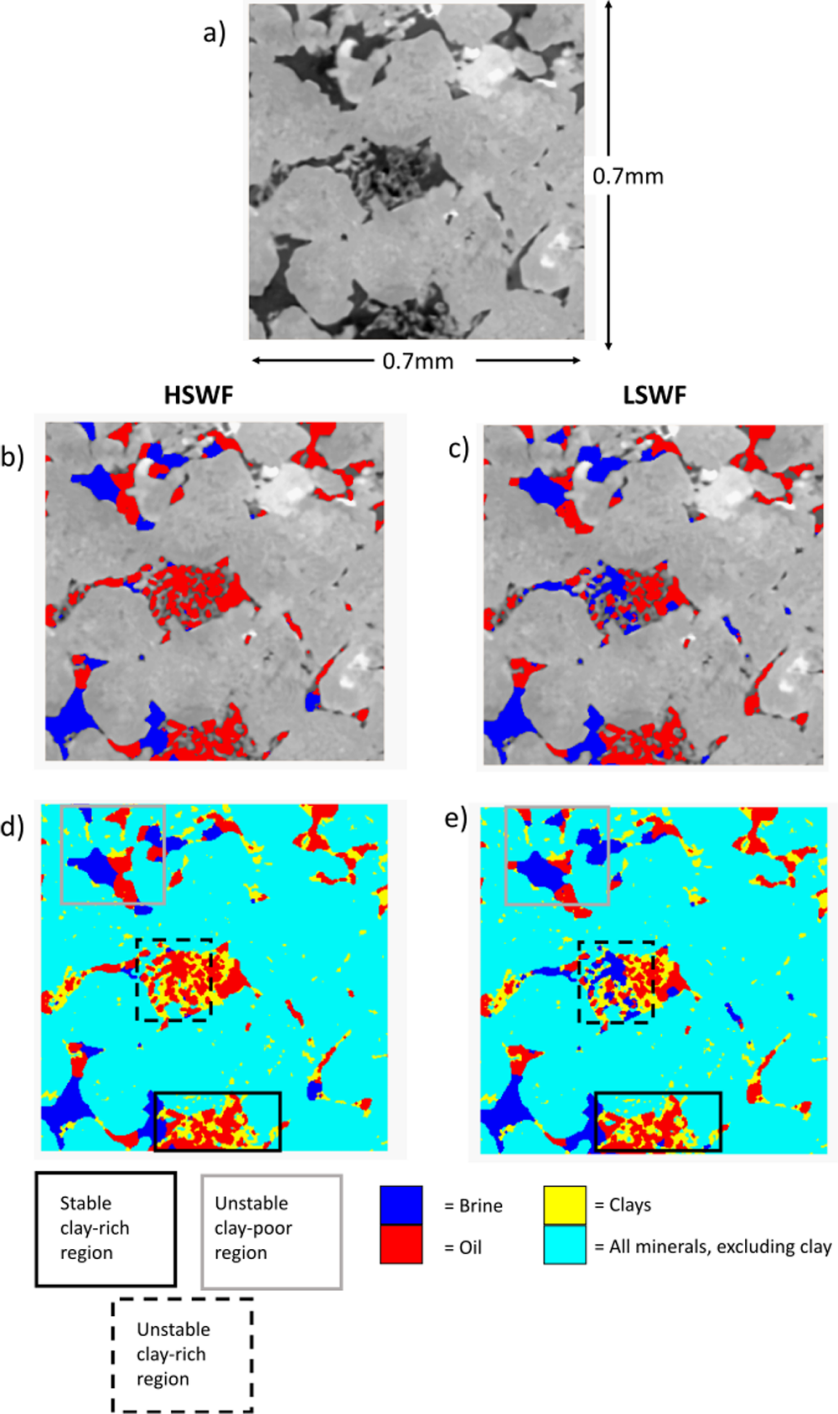
Direct observations of mineralogy and fluid distribution within a subvolume of the Berea sample. (a) Undoped scan of slice through subvolume, (b) raw scan of minerals presented with segmented fluid phases (blue = brine, red = oil) after high-salinity waterflooding, (c) raw scan of minerals presented with segmented fluid phases (blue = brine, red = oil) after low-salinity waterflooding, (d) segmented minerals (turquoise = bulk minerals, yellow = clays) and segmented fluid phases (blue = brine, red = oil) after high-salinity waterflooding, and (e) segmented minerals (turquoise = bulk minerals, yellow = clays) and segmented fluid phases (blue = brine, red = oil) after low-salinity waterflooding. Stable clay-rich areas of the pore space are represented by solid black boxes, unstable clay-rich regions are represented by dashed black boxes, and unstable clay-poor regions are represented by gray boxes.

**Figure 10:**
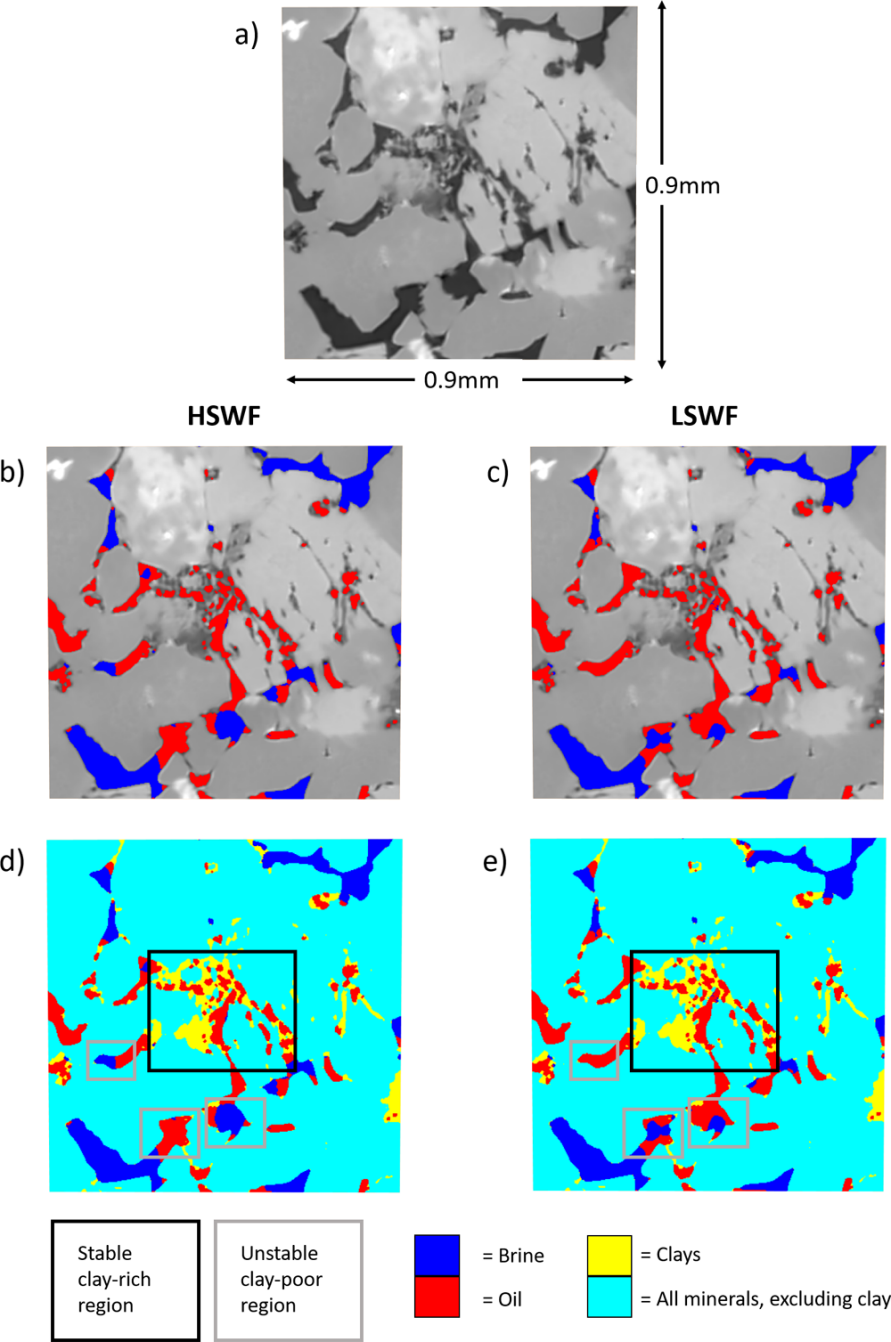
Direct observations of mineralogy and fluid distribution within a subvolume of the Bunter sample. (a) Undoped scan of slice through subvolume, (b) raw scan of minerals presented with segmented fluid phases (blue = brine, red = oil) after high-salinity waterflooding, (c) raw scan of minerals presented with segmented fluid phases (blue = brine, red = oil) after low-salinity waterflooding, (d) segmented minerals (turquoise = bulk minerals, yellow = clays) and segmented fluid phases (blue = brine, red = oil) after high-salinity waterflooding, and (e) segmented minerals (turquoise = bulk minerals, yellow = clays) and segmented fluid phases (blue = brine, red = oil) after low-salinity waterflooding. Stable clay-rich areas of the pore space are represented by solid black boxes, unstable clay-rich regions are represented by dashed black boxes, and unstable clay-poor regions are represented by gray boxes.

**Figure 11:**
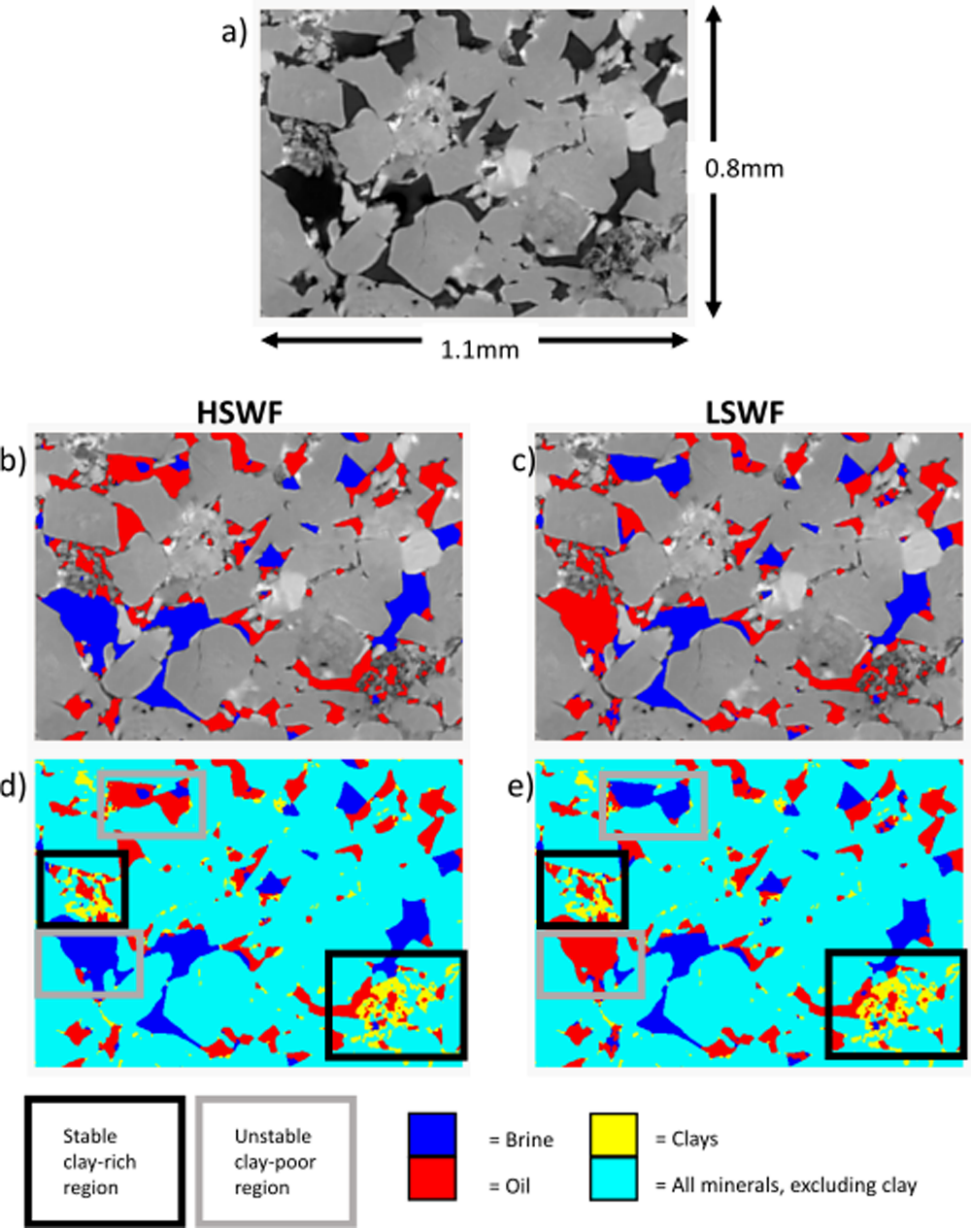
Direct observations of mineralogy and fluid distribution within a subvolume of the Castlegate sample. (a) Undoped scan of slice through subvolume, (b) raw scan of minerals presented with segmented fluid phases (blue = brine, red = oil) after high-salinity waterflooding, (c) raw scan of minerals presented with segmented fluid phases (blue = brine, red = oil) after low-salinity waterflooding, (d) segmented minerals (turquoise = bulk minerals, yellow = clays) and segmented fluid phases (blue = brine, red = oil) after high-salinity waterflooding, and (e) segmented minerals (turquoise = bulk minerals, yellow = clays) and segmented fluid phases (blue = brine, red = oil) after low-salinity waterflooding. Stable clay-rich areas of the pore space are represented by black boxes, and unstable clay-poor regions are represented by gray boxes.

We can directly observe the impact of clays in each sample on the pore-scale fluid dynamics, with significant oil displacement from very clay-rich regions of the Berea sample and no significant oil displacement from very clay-rich pores in the Bunter and Castlegate samples. Figures 9, 10, and 11 show subvolumes of the Berea, Bunter, and Castlegate samples, respectively. In each figure, the undoped scan is presented to show mineralogy together with the fluid configuration after both high- and low-salinity waterflooding.

In all cases, there are large clay aggregates present (highlighted by black boxes). In each case, these regions are completely oil-saturated after high-salinity waterflooding. In the Berea sample, there is significant oil displacement from a clay aggregate (highlighted by a dashed black box) during low-salinity waterflooding. A second clay aggregate is also shown. This second aggregate remains oil-saturated during low-salinity waterflooding. In the Bunter and Castlegate samples, after low-salinity waterflooding, there is no change in the oil configuration within the clay aggregates so that low-salinity brine does not contact the clays at any point throughout the experiment. There are significant fluid occupancy changes in clay-poor pores during low-salinity waterflooding in all samples (highlighted by solid gray boxes). This is consistent with the results presented in Figure 7, which show more significant pore occupancy changes in clay-poor pores than for clay-rich pores. We note that despite the low TDS of the low-salinity water, there is no evidence of clay destabilization or mobilization in any of these samples. This is in terms of either movement of clay particles or flow diversion, resulting from blocking pore throats following mobilization.

### Uncertainty Analysis

We assessed the uncertainty of the measures used in this study by investigating a Berea core that had not been aged and, therefore, remained water-wet. This meant that the oil distribution should not be altered by low-salinity waterflooding. The same image processing analysis workflow was applied to measure saturation distributions and surface area coverage after 12 *PV I* of high-salinity waterflooding, followed by a further 4 *PV I* and 20 *PV I* of low-salinity water injection. We observed very small changes in average saturation, area fraction, and pore occupancy measurements between the three waterfloods, indicating that the image analysis workflow is robust and repeatable. *S*_w_ values of 0.260, 0.256, and 0.254 were recorded after high-salinity waterflooding, 4 PV of low-salinity waterflooding, and 20 PV of low-salinity waterflooding, respectively. This gives a mean value of 0.257, and a standard error of 0.00125. The average *A*_fo_ values for all subvolumes were calculated to be 0.641, 0.646, and 0.646 after high-salinity waterflooding, 4 PV of low-salinity waterflooding, and 20 PV of low-salinity waterflooding, respectively. This gives a mean value of 0.644 and a standard error of 0.00118. These standard errors are an order of magnitude lower than the shifts in *S*_w_ and *A*_fo_ observed during low-salinity flooding in the altered Berea sample. This highlights the robust nature of the image analysis workflows used in this work and gives confidence that the shifts observed in the altered sample reflect physical changes in the samples and not uncertainty in the image analysis techniques.

### Clays as a Low-Salinity Screening Criterion

We observe that the majority of oil displacement in each sample occurs in clay-poor pores. Clay-rich regions of the pore space are preferentially oil-saturated throughout the duration of high- and low-salinity waterflooding. This indicates that clay-rich pores behave differently from clay minerals on planar surfaces, where oil detachment during low-salinity waterflooding is often observed.^29,33–37^ This finding casts doubt on the generally accepted view that oil detachment from clay is the primary mechanism of recovery during low-salinity waterflooding and that clays are essential for favorable recovery during low-salinity flooding.^33–35,67^

While we have shown that clays were not primarily responsible for additional recovery during low-salinity waterflooding in any of the 3 samples, we have observed that clays responded differently in the different samples. In the Berea sample, there was a significant wettability alteration on clay minerals during low-salinity waterflooding, and clay-rich pores contributed 15% of total additional recovery during low-salinity flooding, compared to just 5 and 3% for the Bunter and Castlegate samples, respectively. Below, we briefly describe two hypotheses to explain the differing clay responses between the three samples.

The first potential factor affecting clay behavior is pore structure and connectivity, which is known to impact flow regimes and displacement mechanisms in a range of systems.^71–74,74–77^ Andrews et al.^48^ hypothesized that the connectivity of the largest pores in each of the samples presented here impacted the low-salinity response. We hypothesized that, in the Castlegate sample, the well-connected network of large pores allowed brine to flow easily through the pore space in a stable pathway during secondary high-salinity flooding, so that during subsequent low-salinity waterflooding, the low-salinity water followed the same existing brine pathways, bypassing the remaining oil. We hypothesized that, in the Berea sample and Bunter samples, which have poorly connected networks of large pores over the length scale of the sample, the brine pathway created during high-salinity flooding was broken up due to local and distal oil snap-off events in small and medium pores. As a result, during low-salinity flooding, brine had to displace oil from small and medium pores and throats to connect across the sample, making oil mobilization more likely. This effect is more complex when the mineralogy is considered. In the Berea and Bunter samples, due to this poor connectivity, brine is forced to flow through small pores during low-salinity waterflooding. These are more likely to be clay-rich. As such, it is more likely that brine will come into contact with clays in the Berea sample, allowing for a more significant wettability alteration and oil displacement.

The second potential factor affecting the impact of clay on low-salinity waterflooding is the initial wetting state of each sample. In the Berea sample, both mineral groups are significantly less oil-wetting than in the Bunter and Castlegate samples, which are of comparable initial wetting states (Figure 6). The more oil-wetting nature of the clays in the Bunter and Castlegate samples makes brine invasion into clay-rich regions less favorable, therefore decreasing the likelihood that low-salinity brine will contact clay surfaces and invade clay-rich pores. This is because during waterflooding, the more oil-wetting a pore is, the higher its threshold capillary entry pressure is. The more oil-wetting nature of the Bunter and Castlegate clay aggregates may be the determining factor in preventing brine invasion in clay-rich regions and significant oil production from clay-rich pores.

The results presented here may appear to disagree with the extensive observations of oil detachment from clay minerals on planar surfaces and in micromodels during low-salinity waterflooding.^29,33–37^ This is not the case. We observe that in most cases, it is difficult for low-salinity brine to contact clay minerals during low-salinity flooding, which limits the potential for oil detachment from in situ clay surfaces and additional recovery from clay-rich pores. We have observed in the Berea sample that where low-salinity brine can come into contact with clays, it can cause a significant wettability alteration, resulting in oil detachment and consequently additional oil recovery. An array of complex factors, such as clay distribution, initial wetting state, and pore structure, determine the accessibility of clay aggregates to low-salinity brine and, therefore, the susceptibility to wettability alteration and oil detachment. This is not the case for quartz and feldspar grains, which typically make up the bulk of minerals in sandstones and are present across all of the pore radii. Although quartz and feldspar may be less susceptible to wettability alteration during low-salinity waterflooding, a large body of research has found that significant ion exchange, wettability alteration, and hence oil detachment can still occur on these mineral surfaces.^25–31,78^ Even if this effect is significantly lower than for clays, because quartz- and feldspar-rich pores are more abundant within typical sandstones and are more accessible to invading low-salinity brine, it is likely that most additional oil production in sandstones during low-salinity flooding occurs from quartz- and feldspar-rich pores, as observed here.

We have seen a clear response to low-salinity water within the time scale of these experiments. The duration of low-salinity water injection varied between about 1.5 h for 20 *PV I* through the Bunter and Castlegate cores to 20 h for the Berea core (due to the lower flow rate). Andrews et al.^44^ showed that the pore-scale dynamics changed between 4*PV I* of low-salinity water and 20 *PV I*. There is evidence in the literature that the response can continue to increase over many hours or days. It is possible that further redistribution of oil might have occurred, especially in the smaller pores, which tend to be more clay-rich, if we had continued the experiments for longer. We note, however, that this does not alter our conclusions that most of the oil recovery during low-salinity waterflooding will come from the clay-poor pores, as those hold more of the oil volume.

## Conclusions

In this work, we investigated the impact of a naturally heterogeneous clay distribution on tertiary low-salinity waterflooding. This was achieved by performing flow experiments in Berea, Bunter, and Castlegate sandstone microcores of altered wetting states. The three samples had similar mineralogy, containing 4–10 wt % clay, but had different pore topologies and geometries.

In all three samples, oil was trapped at relatively high saturations after high-salinity waterflooding, with *S*_o_ values of 0.62, 0.57, and 0.61 in the Berea, Bunter, and Castlegate samples, respectively. In all samples, a modest low-salinity response was observed, with additional recoveries of 3, 4, and 1 percentage points during low-salinity waterflooding in the Berea, Bunter, and Castlegate samples, respectively.

We used surface area fractional coverage analysis to observe in situ mineral-specific wettability changes that occurred due to low-salinity waterflooding. In all samples, clay minerals were significantly more oil-wetting than nonclay minerals. In the Berea sample, we observed the largest wetting alteration on clays, with a significant shift to more water-wetting conditions for both clays and nonclay minerals. In the Bunter sandstone, there was a wettability alteration toward more water-wetting conditions for both mineral groups; however, this was the largest for nonclay minerals. In the Castlegate sample, no systematic wettability alteration was observed for either mineral group.

For all samples, there was an increase in brine saturation in small clay-poor pores during low-salinity waterflooding, consistent with a wettability alteration to more water-wet conditions. This was the most significant in the Berea and Bunter sandstones. In all samples, the majority of oil displacement occurred in clay-poor pores, with very few oil displacement events in small clay-rich pores in the Bunter and Castlegate samples. Clay-rich pores accounted for only 15, 5, and 3% of total additional recovery in the Berea, Bunter, and Castlegate samples, respectively.

For very clay-rich pores (pores with >90% clay coverage), *S*_o_ was >0.95 after high-salinity waterflooding for all samples. There was no significant change in *S*_o_ in these pores in the Bunter and Castlegate samples during low-salinity waterflooding; however, we observed a 5 percentage point decrease in *S*_o_ in very clay-rich pores in the Berea sample. This behavior was observed directly in images of the pore space, with clay aggregates remaining oil-saturated throughout the experiment in Bunter and Castlegate samples but with some low-salinity brine invasion into clay aggregates in the Berea sample. It is likely that in the Bunter and Castlegate samples, clay-rich regions were so strongly oil-wet that low-salinity brine could not enter those pores and, therefore, could not contact significant volumes of clays. Consequently, only small amounts of oil detachment could occur.

The presence of clays does not appear to be a good indicator that low-salinity waterflooding will improve recovery in sandstones. Clays can positively impact production in areas where they can be accessed by low-salinity brine, as in the Berea sample. However, the presence of clays does not guarantee improved production, as brine may not be able to contact the majority of the clay mineral surfaces. These observations will apply to secondary and tertiary low-salinity waterflooding because they apply to pores that were never accessed by the high-salinity waterflood. Further research should concentrate on quantifying the accessibility of clay-rich regions, considering pore structure and connectivity, initial wetting state, and the distribution of clays within samples, and developing this as a measure to help predict low-salinity response.
